# Mycobacterial skin infection

**DOI:** 10.1097/QCO.0000000000000820

**Published:** 2022-01-19

**Authors:** Giulia Gardini, Natalia Gregori, Alberto Matteelli, Francesco Castelli

**Affiliations:** a University Department of Infectious and Tropical Diseases, University of Brescia and ASST Spedali Civili, Brescia, Italy; b WHO Collaborating Centre on TB/HIV collaborative activities and for the TB Elimination Strategy, University of Brescia, Brescia, Italy; c UNESCO Chair Training and Empowering Human Resources for Health Development in Resource-Limited Countries, University of Brescia, Brescia, Italy; d ESCMID Study Group for Infections in Travelers and Migrants (ESGITM), Basel, Switzerland

**Keywords:** Buruli ulcer, leprosy, nontuberculous mycobacteria, tuberculosis

## Abstract

The aim of this article is to review the most recent evidences concerning mycobacterial skin infections, limiting the period of literature research to 2020--2021.

**Recent findings:**

Mycobacterial skin infections include a heterogeneous group of cutaneous diseases.

Cutaneous tuberculosis is usually the result of hematogenous dissemination or spread from underlying foci and it must be distinguished from tuberculids, resulting from the immunological reaction to *Mycobacterium tuberculosis* antigens. Leprosy prevalence was drastically reduced after introduction of multidrug therapy in the 1980 s, but cases are still reported due to underdiagnosis, and animal and environmental reservoirs. Recent advances concentrate in the diagnostic field. Specific guidelines for the treatment of nontuberculous mycobacteria skin infections are missing and surgical procedures may be required. Prognosis is better as compared to nontuberculous mycobacteria lung disease. Rapid laboratory-confirmed diagnosis of Buruli ulcer may be achieved by the IS2404 PCR. Among new drugs, telacebec is promising in terms of potency, shorter duration and tolerability in animal studies. A clinical trial in humans is planned.

**Summary:**

Mycobacterial cutaneous lesions are nonpathognomonic and clinical suspicion must be confirmed by culture or molecular detection. Long-course multidrug treatment is required based on susceptibility tests. Surgical intervention may also be required. Rehabilitation and psychosocial support reduce long-term physical and mental consequences mostly in Buruli ulcer and leprosy.

## INTRODUCTION

The *Mycobacterium* genus, the only entity within the *Mycobacteriaceae* family (order *Actinomycetales*, phylum Actinobacteria), consists of a distinct group of aerobic, nonmotile, nonspore-forming, Gram-positive bacilli. They are characterized by the slow rate of growth and the high content of mycolic acids within the cell wall, which makes them resistant to alcohol and acid rinsing. 

**Box 1:**
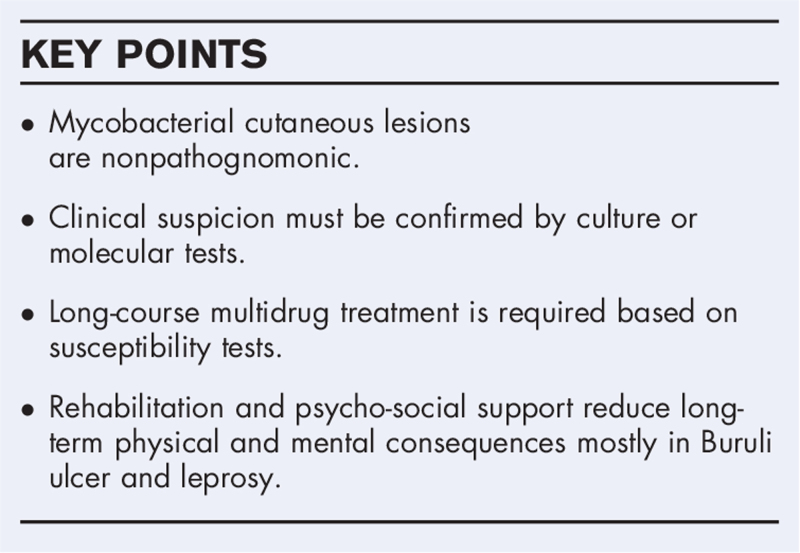
no caption available

Skin and soft tissue infection (SSTI) by *Mycobacterium tuberculosis* complex is rare but remains a potential threat in developing regions. Cutaneous tuberculosis (CTB) must be differentiated from tuberculids, based on the demonstration, in the former, of vital mycobacterial bacilli in biopsies. Among 10 million TB cases estimated globally by WHO in 2019 [1], extra-pulmonary localization accounted for 16% and CTB for less than 2% of all extra-pulmonary cases [1,2].

**FIGURE 1:**
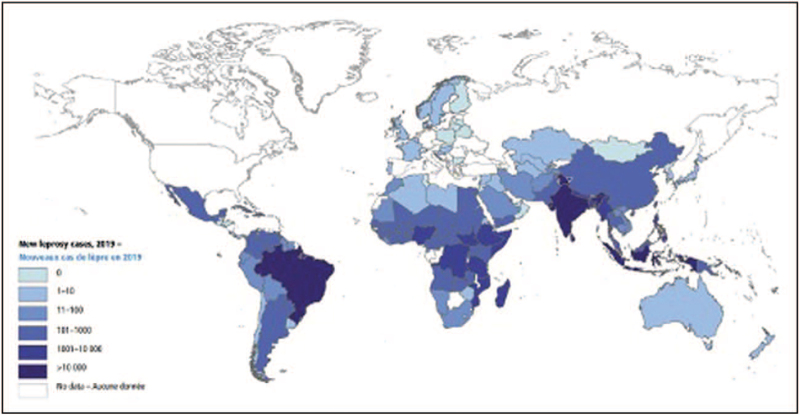
New leprosy cases in 2019 (Source: WHO, Weekly Epidemiological Record No 36, 2020, 95, 417–440).

In 2001, leprosy was declared eliminated as a public health problem globally (prevalence rate < 1/10 000 population) [3]. However, it still remains endemic in some areas of the world (Fig. 1). Stigmatization and discrimination against affected people continue to represent a barrier for leprosy elimination.

Nontuberculous Mycobacteria (NTM) are ubiquitous microorganisms, divided into hundreds of species, with variable relationship with humans. Differently from *M. tuberculosis*, they may simply colonize humans. Skin infections are associated typically to *M. marinum* and few other species. NTM are clearly neglected: the 2007 ATS/IDSA guidelines [4] dedicated only a short paragraph to skin involvement, describing causative species and how to prevent healthcare associated disease. The ATS/ERS/ESCMID/IDSA Clinical Practice Guideline of 2020 [5] updates treatment recommendations only of pulmonary NTM (pNTM).

Buruli ulcer is a cutaneous and subcutaneous often disfiguring disease caused by *M. ulcerans*. WHO first declared Buruli ulcer a neglected emerging tropical disease in 1998 and launched a Global Buruli Ulcer Initiative [6].

Differently from other NTM, this disease has gained global attention possibly due to a higher incidence within endemic areas and in infant populations, and the psychosocial impact due to functional impairment and stigma.

Among clinical diseases due to mycobacteria, SSTIs continue to be poorly investigated despite their impact on persons’ quality of life and the evidence of increasing incidence in recent years [7,8].

We have performed a nonsystematic revision of the literature, limited to the period from January 2020 to August 2021, searching for epidemiological, clinical, diagnostic and treatment publications on cutaneous manifestations in tuberculosis, leprosy, Buruli ulcer and NTM infections.

## MYCOBACTERIAL SKIN INFECTIONS

### Cutaneous tuberculosis

*M. tuberculosis*, *M. bovis* and, less commonly, the bacillus of Calmette-Guerin (BCG) (an attenuated strain of *M. bovis*) are the etiologic agents of CTB.

Development of CTB depends on load and pathogenicity of the infecting strain, the route of infection, the patient's prior sensitization to tuberculosis and state of the patient's cell-mediated immunity [9]. Head and neck are the most involved sites (about 90% of CTB patients) with predilection of nose and cheek. Ear involvement is very rare with only a few case reports in the literature [10].

The skin can be affected by *M. tuberculosis* through inoculation from an exogenous source, spread from a contiguous source and haematogenic dissemination [9,11].

’TB chancre’ identifies the form that results from the entry of mycobacteria through skin or mucosa lesions. It has been largely correlated to occupational exposure in healthcare settings due to injuries with poorly sterilized equipment, but it is also associated with highly risky procedures as tattooing, circumcision, piercing and acupuncture [12].

*TB verrucosa cutis* is the consequence of re-exposure to mycobacteria in individuals with preexisting immunity to *M. tuberculosis*. It is observed more commonly in children and young adults because of their efficient lymphatic drainage, and their greater risk for traumatic injuries [13]. This rare entity might be confused with diseases such as leishmaniasis, sporotrichosis, nocardiosis, atypical mycobacteria (*M. marinum*), pyogenic infections (*Staphylococcus aureus*, *Streptococcus spp*) and deep fungal infections [14^▪^].

**FIGURE 2:**
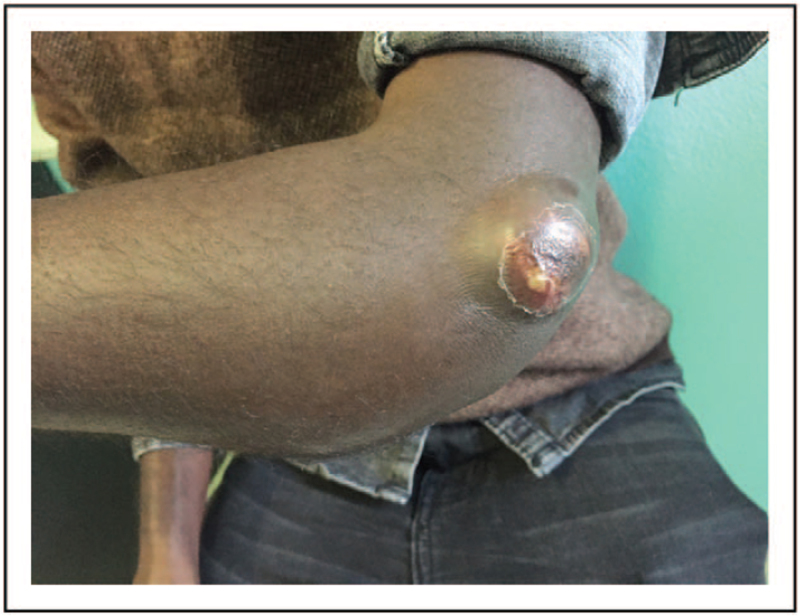
Bone and cutaneous tuberculosis in Ethiopian migrant.

**FIGURE 3:**
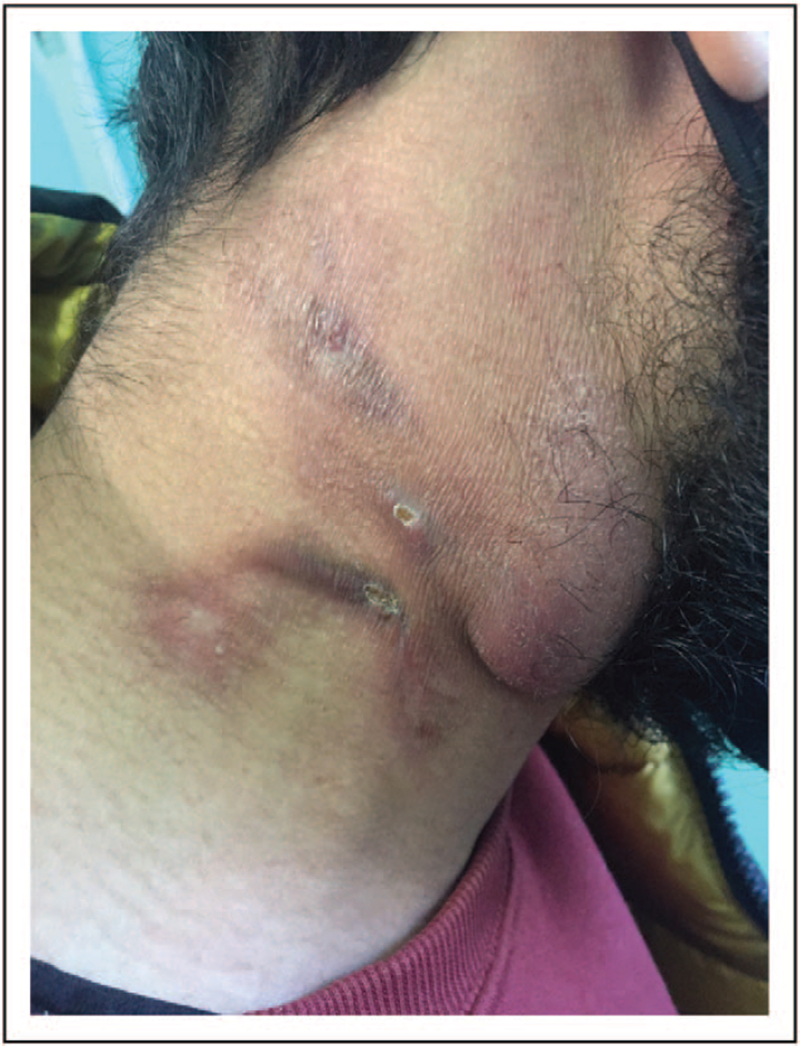
Lymph nodal and cutaneous tuberculosis in a man from Morocco.

CTB from an endogenous source, also known as ‘scrofuloderma’, originates from a contiguous tuberculosis focus (e.g. lymphadenitis, osteomyelitis, epididymitis), which spreads to the overlying skin leading to local tissue destruction (Figs. 2 and 3). Neck, axillae, groin and chest are the most commonly involved sites [15]. Orificial TB (or *TB cutis orificialis*) is a rare postprimary CTB caused by autoinoculation of mycobacteria in middle-aged to elderly patients with advanced forms of lung, intestinal or genitourinary TB [16]. Lesions appear more frequently in oral mucosa, but the perianal area can also be affected [16]. Differential diagnoses of perianal ulcers include inflammatory processes (e.g. Crohn's disease), neoplasms, sarcoidosis and sexually transmitted diseases [16].

Haematogenous or lymphatic dissemination accounts for the majority of cutaneous cases of TB. Among them, *lupus vulgaris* is a chronic, progressive, postprimary form of CTB, following a regional lymphatic or blood stream spread in individuals with moderate or high level of TB immunity [17^▪^]. Various clinical forms are observed: papular, nodular, with plaques, vegetative, ulcerative, mutilating, hypertrophic, atrophic, tumour-like type [17^▪^]. The buttocks and the extremities are usually involved in tropical and subtropical settings, face and neck in the West [17^▪^]. Ramesh *et al.*[10] recently described an Indian case series of *lupus vulgaris* affecting the pinna [10]. Family history of *lupus vulgaris* has been described in high endemic areas [17^▪^]. Main differential diagnosis include leprosy, sarcoidosis, discoid lupus, leishmaniosis [17^▪^]. Acute miliary skin TB and gummous TB are more commonly seen in patients with immunosuppressive disorders (i.e. AIDS, users of tumour necrosis factor alpha antagonists) [18].

Tuberculids are a form of hypersensitivity reaction to mycobacterial antigens in cutaneous blood vessels in patients with high immunity to *M. tuberculosis*. Their clinical appearance is heterogeneous: papulonecrotic lesions, *lichen scrofulosorum*, reported as the most common tuberculid lesion in children [19^▪^], and *erythema induratum* of Bazin (EIB), the latter is the most frequent form of tuberculid, commonly seen on the posterior part of the legs [20^▪^].

Also, *BCG vaccination or immunotherapy* has been related to local cutaneous complications including localized abscess, scrofuloderma, *lupus vulgaris* and nonhealing ulcers.

Disseminated cutaneous tuberculosis is rarely reported both in immunosuppressed and immunocompetent children receiving the vaccine at birth [21].

As CTB is rare and with no typical manifestations, the diagnosis is challenging and easily mistaken with other skin diseases [2]. Histopathology is not specific, as it shows granulomatous lesions both in true infections and in immunological reactions. The sensitivity of Ziehl-Nielsen AFB stain is low in CTB [2,22]. Thus, culture and amplification of *M. tuberculosis* DNA by PCR in skin biopsies are the gold standard for diagnosis [11]. *M. tuberculosis* cannot be found in smear nor cultured from tuberculids lesions, although mycobacterial DNA may occasionally be detected by PCR [2,19^▪^].

In case of high index of suspicion but lack of microbiological confirmation, empirical tuberculosis treatment is warranted [10].

Cutaneous TB is treated using the WHO standard regimen: 2 months of rifampicin, isoniazid, pyrazinamide and ethambutol followed by 4 months of rifampicin and isoniazid [23]. Recently, in-vivo pharmacokinetic studies have shown promising results from transdermal delivery of rifampicin as strategy for the treatment of systemic and cutaneous tuberculosis [24^▪▪^]. TB treatment is not recommended for tuberculids lesions. A retrospective study of 22 patients with EIB found that the rate of recovery was 75% in patients receiving TB treatment and 87.5% in patients without TB treatment [20^▪^].

In addition to chemotherapy, surgical intervention may be required [9,25].

### Leprosy (or Hansen's disease)

Leprosy, or Hansen's disease, is a chronic mycobacterial infection caused by *M. leprae* or the recently discovered *M. lepromatosis.*

The upper respiratory tract (in particular the nose) and skin lesions are considered the main source of transmission and acquisition of *M. leprae*[26]. Solid evidence exists on the increased risk of infection in household contacts of leprosy patients [27,28^▪▪^]. Mother-to-child transmission has also been described [27]. TNF alpha inhibitors (infliximab, etanercept, adalimumab) have been proposed as facilitators of leprosy reactivation [29].

Although the incidence of leprosy has declined by 95% since the introduction of multidrug therapy (MDT) in the 1980 s, it has plateaued in the last decade [30,31^▪▪^]. The current stagnation in leprosy incidence has been attributed both to the large number of undetected cases, who contribute to furthering human to human transmission, and to the role of animal and environmental reservoirs [28^▪▪^,^31^^▪▪^,^32^]. Armadillo species and red squirrels were found to be wildlife reservoirs of *M. leprae*[31^▪▪^,^33^]. Viable *M. leprae* has also been detected from environmental sources (water and soil) in Indonesia, India, Brazil and Bangladesh [34]. Recent studies have shown a potential role of vectors like kissing bugs and free-living amoebas [28^▪▪^].

**FIGURE 4:**
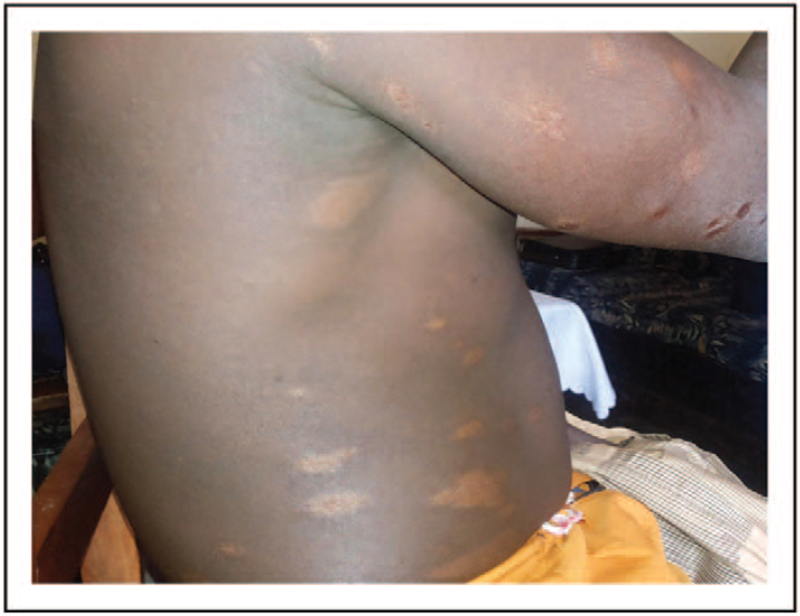
Tuberculoid leprosy lesions on the trunk (Courtesy of Damien Foundation Burundi).

**FIGURE 5:**
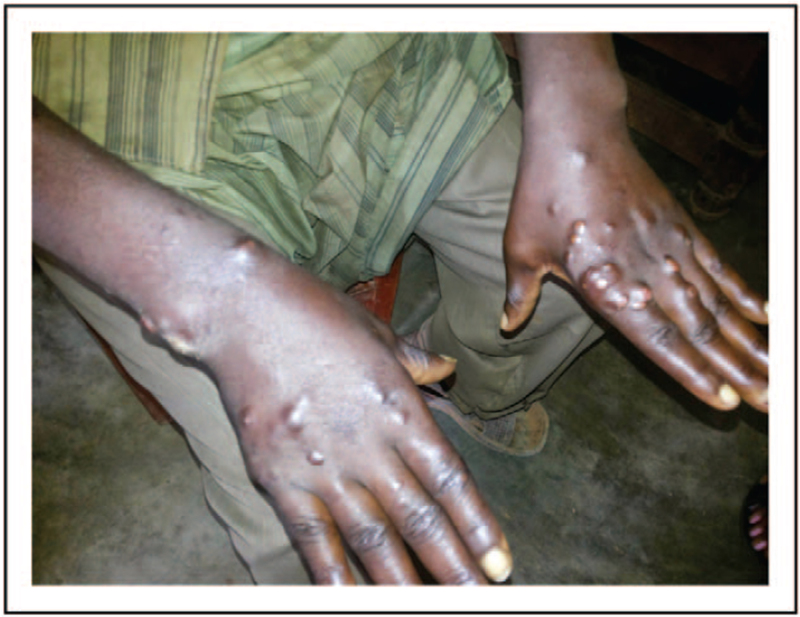
Lepromatous leprosy lesions on the hands (Courtesy of Damien Foundation Burundi).

The clinical presentation is determined by the microorganism's tropism for skin and peripheral nerves (preferentially Schwann cells) and its variability by the genetic background and the immune response of the host [27,33,35^▪^]. Murine models have shown that the Th1 cascade, with IL-2 and IFN-gamma production, may be responsible for tuberculoid lesions (Fig. 4). On the contrary, Th2 cytokine cascade (IL-4 and IL-5) is responsible for lepromatous lesions (Fig. 5) [35^▪^].

Pure neuritic leprosy without skin lesions is common in Nepal, Brazil and India (4–18% of Indian leprosy cases). In such cases, mononeuritis is the most common presentation [36], especially in older patients, suggesting the effectiveness of national control measures against child leprosy [31^▪▪^].

Suspicion of the disease may be triggered, in an endemic context, by the onset of sensory impairment, paraesthesia, motor involvement, nerve enlargement. The diagnosis is based on skin and nerve biopsies for histopathology and PCR, or by slit skin smears for AFB detection through Ziehl-Neelsen staining [27,36]. Calculating the bacillary index on a biopsy allows to establish whether the disease is multi or paucibacillary and determines the treatment.

Rapid diagnostic tests based on the detection of an IgM antibodies response against *M. leprae* specific antigens have shown utility in detecting multibacillary patients but limited sensitivity in paucibacillary ones. The most studied serological platforms are those directed to phenolic glycolipid I (PGL–I) and NDO-LID antigens [27,36–38]. Intradermal skin testing, based on delayed type hypersensitivity (DTH) reaction to various antigen preparations, may reach the sensitivity required for a *M. leprae* screening/surveillance tool. New *M. leprae* skin tests based on candidate antigens are under investigation. The submerged proportion of individuals with latent disease is a main target for serological diagnosis. Combination of two biomarkers of leprosy as serology (positive anti PGL-I via ELISA essay) and *M. leprae* DNA detection (positive PCR from earlobe skin smears) is a promising approach in detecting people with latent leprosy [30]. Moreover, elaboration of host immune profiling of household contacts through molecular methods may help in identifying biomarkers associated with *M. leprae* exposure or asymptomatic infection [39].

Multidrug therapy is the cornerstone of leprosy treatment, consisting in the WHO recommended three-drug regimen (rifampicin as well as clofazimine and dapsone) for 6 months in paucibacillary forms and for 12 months in multibacillary forms [40]. As MDT is not able to reverse nerve damage, a timely treatment of leprosy reactions with corticosteroids is recommended to control acute inflammation. However, the exact duration of steroid treatment has not been established.

Although BCG vaccination confers some level of protection [41], no vaccine is currently available that specifically targets *M. leprae*. Exposed individuals may effectively receive chemoprophylaxis with a single dose of rifampicine (SDR) 600 mg per day (adults and children >2 years of age) [42]. A new subunit vaccine containing recombinant polyprotein LEP-F1 (LepVax) started the development programme: a single-centre Phase 1, open-label clinical trial provided encouraging safety and immunogenicity results [43^▪▪^].

### Nontuberculous mycobacteria skin infection

Potentially all NTM species can cause skin infections; however, for the slow growing mycobacterium (SGM) *M. marinum*, the skin is the typical site of infection, and the rapidly growing mycobacteria (RGM) *M. fortuitum*, *M. abscessus* and *M. chelonae* may also give cutaneous disease. MAC complex [44] and less pathogenic NTM may also determine SSTIs, as reported for *M. haemophilum*[7,45,46], *M. gordonae*[47] and *M. agri*[48].

Skin is the second more common site of NTM infection both in immunocompromised and immunocompetent individuals, following lung disease in adults and lymphadenopathy in children.

NTM are ubiquitous organisms, thanks to their resistance to high temperature, disinfectants and limited need for nutrients and oxygen. Several factors influence the frequency of NTM skin infections in different areas, such as frequency and quality of medical invasive procedures, common activities and habits (e.g. gardening, farming, swimming, fishing). Climatic and environmental conditions may also play a role: paediatric skin infections due to *M. heamophilum* increased in tropical coastal areas in Australia after a major flooding [49].

Skin infection usually follows inoculation through contact of damaged skin with environmental niches such as tap water or through invasive medical procedures. Pavli *et al*. [50^▪^] reviewed the studies from 2010 and 2019 reporting medical tourism-related infections, in particular after cosmetic surgery and transplantation, and NTM emerged as a significant cause. Human-to-human transmission has not been postulated in mycobacterial skin infections. Animals may be colonized and infected by NTM [51–54], but animal to human transmission has not been demonstrated, except from *M. marinum* that can be transmitted to humans by contact with aquatic animals.

**FIGURE 6:**
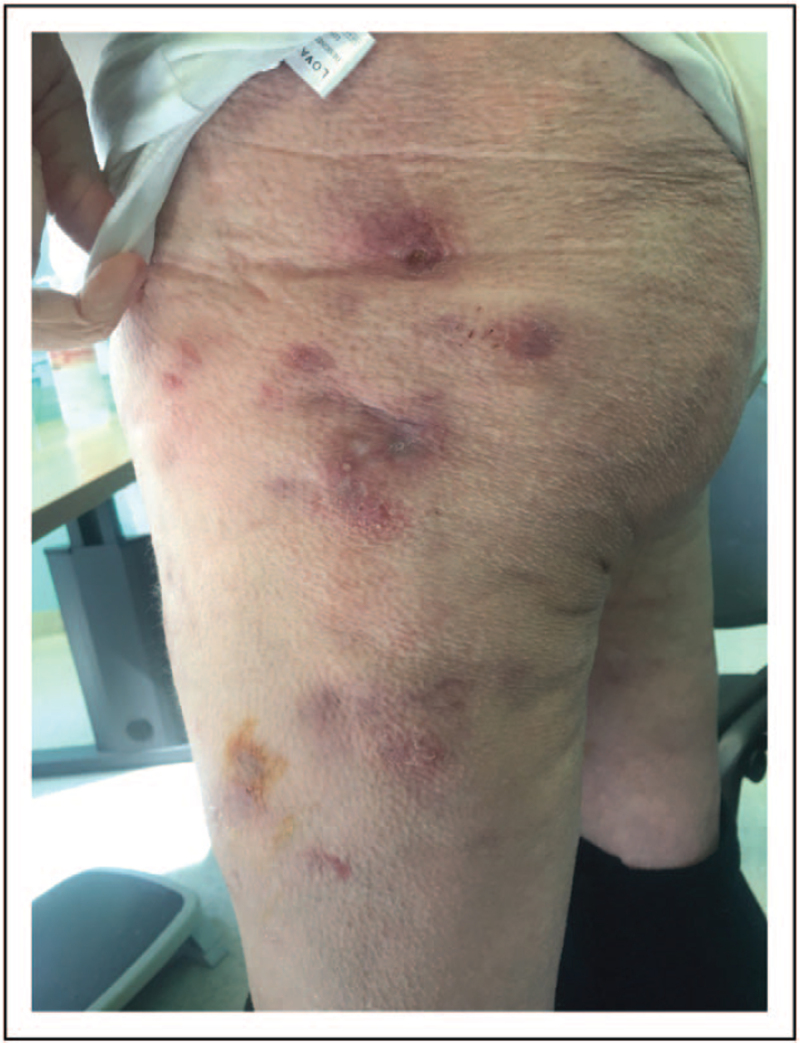
Disseminated skin infection by *M. chelonae* in immunocompromised woman.

NTM skin lesions have no pathognomonic characteristics. Clinical manifestation and incubation period are heterogeneous depending on modality of mycobacterial acquisition, bacterial load and virulence and host immune status [55]. Immunocompromised individuals are more susceptible to less pathogenic NTM (i.e. *M. haemophilum*) and dissemination (cutaneous and/or subcutaneous and/or systemic) (Fig. 6).

Diagnosis requires culture for mycobacteria. *M. haemophilum, M. genavense, M. avium* subsp. paratuberculosis and *M. ulcerans* require specific supplements in culture media. In-vitro antimicrobial susceptibility testing is recommended.

Specific guidelines for the treatment of NTM skin infections are missing. Multidrug regimens are recommended, as for pNTM infections (three in-vitro active agents for MAC and *M. abscessus*; two for *M. chelonae*, *M. fortuitum* and *M. marinum*), but duration is generally shorter (6–12 months for MAC and *M. abscessus*; at least 4 months for *M. chelonae* and *M. fortuitum*; 3–4 months or 1–2 months after resolution of symptoms for *M. marinum*) [4]. Chirasuthat *et al.*[7] reported a higher rate of relapse [odds ratio (OR) 65.86; *P* = 0.02] in a cohort of 88 patients with cutaneous infections by *M. abscessus* (61.4%), *M. haemophilum* (10.2%) and *M. marinum* (8.1%) if treated with less than three antibiotics. *M. abscessus* remains a challenge for clinicians due to its large spectrum of virulence and resistance mechanisms [56^▪^]. A few case reports described the use of bedaquiline to treat cutaneous NTM disease [57,58]. The use of omadacycline [59], tedizolid [60^▪^] and linezolid [61] was also reported in single cases.

Additional surgery may be needed for the management of cutaneous NTM [62].

Prognosis for cure is better as compared to pNTM infection. Hannah *et al*. [62] reported complete healing in 83.9% of cases (47 out of 56).

Behavioural precautions to limit environmental NTM exposure are recommended only for solid organ transplant recipients, due to lack of evidence in terms of harms and benefits [63]. The existing guidelines strongly recommend not to use tap water for healthcare and hygiene procedures [4].

### Buruli ulcer

Buruli ulcer is caused by *M. ulcerans*, whose ancestor is *M. marinum*. *M. ulcerans* grows at temperature between 28°C and 33°C and its main virulence factor is the plasmid-encoded toxin mycolactone that downregulates host innate and Th1 response and induces tissue damage determining host cell apoptosis [64]. Foulon *et al*. [65] emphasized the pro-inflammatory activity of the toxin in later stages of the infection that leads to destructing lesions.

West Africa and Australia are endemic regions for Buruli ulcer. Sepulcri *et al.*[66] described recently the first case of the disease in a traveller returning from Madagascar. Surveillance of Buruli ulcer cases is a main target for WHO [67].

Global annual incidence of Buruli ulcer varies and in 2020 a reduction of about 1000 notified cases compared to the previous year has been reported [64]. However, this might be associated to the limited active screening campaigns during COVID-19 pandemic. Temperature increase may also negatively affect environmental reservoir (reduced water basins) and mycobacterial survival [68^▪▪^].

Aquatic environments may represent the natural niches for *M. ulcerans*. Aquatic insects in West Africa, and mosquitoes in Australia have been proposed as vectors in Buruli ulcer, but their role remains unclear [69^▪▪^]. Australian opossums have been investigated as animal reservoirs, as they are affected by Buruli ulcer and *M. ulcerans* DNA has been detected in their faeces [69^▪▪^]. A few cases of Buruli ulcer have been described also in other mammals in Australia, but animal-to-human transmission has not been demonstrated yet.

The exact modality of *M. ulcerans* transmission and contributing actors are still objects of research and debate. Contact between wounds and contaminated water may generate skin infection, as suggested by the detection of *M. ulcerans* DNA in aquatic habitats, the increased Buruli ulcer incidence during wet and rainy seasons, the closeness of water habitats as predictor for Buruli ulcer occurrence in spatial models, and results of prevention case--control studies [69^▪▪^]. Most environmental studies have used molecular probes; therefore, they cannot inform about viability and transmissibility of the bacteria. Human-to-human transmission has not been demonstrated.

Genome-wide association studies demonstrated the presence of genetic susceptibility factors that may explain familial cluster of Buruli ulcer and heterogeneity of clinical patterns and prognosis [70–72]. Fevereiro *et al.*[73] identified the extreme ages and HIV coinfection as risk factors for Buruli ulcer. HIV-positive status may also determine a more aggressive form of disease and a poorer treatment outcome [64].

**FIGURE 7:**
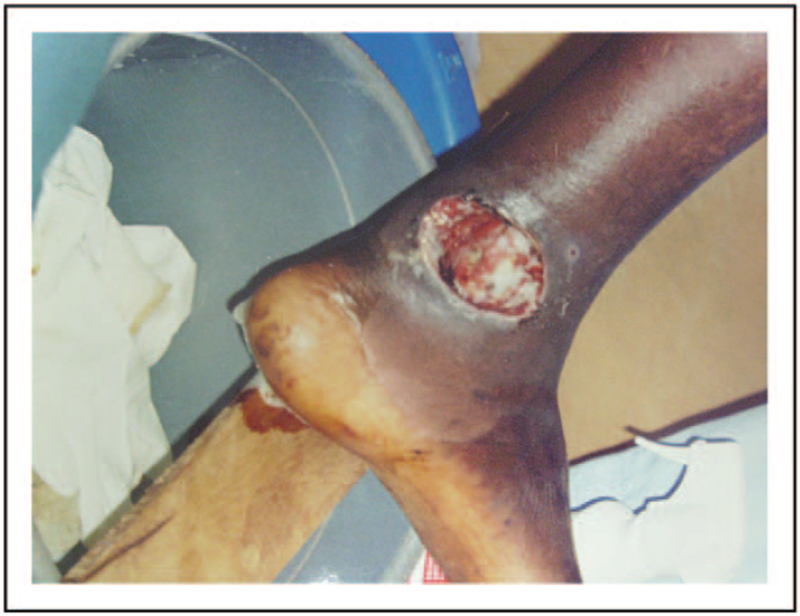
Buruli ulcer in a man from Ivory Coast (Courtesy of Dr Antonella Bertolotti).

The first lesion may appear at the site of inoculation as a nodule, plaque or oedema to evolve afterwards in a disfiguring often painless ulcer with yellowish surface, red based and surrounded oedema [74] (Fig. 7). Limbs are mostly affected. The clinical pattern distinguishes three categories according to increasing severity and involvement of deeper tissues [64]. During treatment, a paradoxical worsening of the lesion may be observed.

Social stigma segregates patients and their family, due to low awareness of transmission modality. Depression and anxiety may affect patients and may persist even after recovery [75,76].

A mobile application may be used in cases with possible disease [74]. Prompt laboratory-confirmed diagnosis is a main goal of WHO 2030 targets [77]. A rapid diagnostic tool is IS2404 PCR. The test is supported by WHO in African endemic regions to limit misdiagnosis and diagnostic delay [64]. Direct microscopy, culture and histopathology require more time and laboratory experience.

Recommended treatment is rifampicin 10 mg/kg/day with clarithromycin 7.5 mg/kg twice daily for 8 weeks [64,78]. Moxifloxacin 400 mg/die may substitute clarithromycin and is commonly used in Australia [79]. Combination with streptomycin is also effective, but less tolerated [78,80]. Treatment failure is uncommon; O’Brien *et al*. [79] identified in an Australian cohort weight more than 90 kg (*P* < 0.001), male sex (*P* = 0.02), immune suppression (*P* = 0.04) and rifampicin-clarithromycin regimen instead of rifampicin-fluoroquinolone (*P* = 0.05) as risk factors for failure.

Among new drugs, telacebec is promising in terms of potency, shorter duration (2 weeks) and tolerability in animal studies [64,81–83]. In January 2021, telacebec was recognized as an orphan drug by US Food and Drug Administration. A clinical trial in humans is being planned [64].

Surgical intervention may be required in selected cases, always in conjunction with chemotherapy [78]. Prolonged wound medications, management of lymphoedema and functional rehabilitation are often necessary, as ulcers require months to heal [84].

Educational activities, periodic screening campaigns, diffusion of molecular rapid diagnostic tests and free and integrated treatment are WHO-sustained tools to limit the psycho-social-economic burden of Buruli ulcer in endemic regions [77].

## CONCLUSION

Cutaneous mycobacteriosis remain underdiagnosed and undertreated due to economic reasons, low awareness and stigmatization. A reduction in the burden of these diseases requires a comprehensive approach, including community education in endemic areas, increased understanding of animal and environmental reservoirs, diffusion of rapid diagnostic tools, universal accessibility to free multidrug treatments and a multidisciplinary approach.

## Acknowledgements

*None.*

### Financial support and sponsorship

*None.*

### Conflicts of interest

*There are no conflicts of interest.*

### Disclaimer

*The author is responsible for the views contained in this article and for opinion expressed therein, which are not necessarily those of UNESCO and do not commit the organization.*

## REFERENCES AND RECOMMENDED READING

Papers of particular interest, published within the annual period of review, have been highlighted as:

▪ of special interest
▪▪ of outstanding interest
